# Haemodynamic Responses to Tracheal Intubation Using Propofol, Etomidate and Etomidate-Propofol Combination in Anaesthesia Induction

**DOI:** 10.15171/jcvtr.2015.30

**Published:** 2015-11-26

**Authors:** Özgür Yağan, Nilay Taş, Ahmet Küçük, Volkan Hancı, Bülent Serhan Yurtlu

**Affiliations:** ^1^ Ordu University, School of Medicine, Department of Anesthesiology, Ordu, Turkey; ^2^ Harran University, School of Medicine, Department of Anesthesiology, Sanlıurfa, Turkey; ^3^ Dokuz Eylül University, School of Medicine, Department of Anesthesiology, Izmir, Turkey

**Keywords:** Propofol, Etomidate, Endotracheal Intubation, Hemodynamic

## Abstract

***Introduction:*** The aim of this study was to measure the haemodynamic responses to a etomidate-propofol combination used for anaesthesia induction and to compare the haemodynamic responses with the separate use of each drug.

***Methods:*** The patients were randomly divided into three groups as group P (n = 30, propofol 2.5 mg kg^-1^), group E (n = 30, etomidate 0.3 mg kg^-1^) and group PE (n = 30, propofol 1.25 mg kg^-1^ + etomidate 0.15 mg kg^-1^). For each patient, the times of measurement of the heart rate (HR) and mean arterial pressure values were defined as baseline, after the induction, before the intubation, immediately after the intubation and 1, 2, 3, 4, 5 and 10 minutes after the intubation.

***Results:*** In all 3 groups, a significant decrease in MAP values were seen at T2 and T3 compared to the baseline values, and this decrease was greater in group P compared to that in group E and PE (*P* < 0.001, *P* < 0.01). A significant increase was seen in all 3 groups in the mean arterial pressure (MAP) value at T4 after the intubation. When the groups were compared with each other, this increase was greater in group E than in the other two groups (with group P, *P* < 0.001; with group PE, *P* < 0.01).

***Conclusion:*** Etomidate-propofol combination may be a valuable alternative when extremes of hypotensive and hypertensive responses due to propofol and etomidate are best to be avoided.

## Introduction

In all methods used for anaesthesia induction, it is aimed to preserve the haemodynamic balance and to provide optimal conditions for the patient by reducing side effects. However, when intravenous induction drugs are used as a single hypnotic agent, haemodynamic side-effects are frequently observed.^1,2^

Propofol is a frequently-used intravenous anaesthetic with an effect of rapid onset and short duration. During anaesthesia induction with propofol, often seen side effects are injection pain and a fall in arterial blood pressure.^3,4^ Etomidate is a hypnotic agent with minimal effects on the cardiovascular system. It does not cause histamine expression and has no analgesic properties. Etomidate’s side-effects are primarily injection pain, myoclonus, superficial thrombophlebitis and a high incidence of nausea and vomiting.^3^ Previous studies have also reported that etomidate did not prevent the sympathetic response to laryngoscopy and intubation at a sufficient level.^3,5^

The hypothesis of this study was that with the use of average doses of etomidate and propofol together, the haemodynamic deterioration after the anaesthesia induction and endotracheal intubation would decrease. With the aim of testing this hypothesis, consecutive doses were used within the permitted limits in the clinical use of propofol and etomidate in anaesthesia induction, and the effects on the haemodynamic response to intubation were measured. Primary objective was the comparison of the haemodynamic changes created with the etomidate-propofol combination and the sole use of each drug. Secondary aims were defined as the incidence of injection pain and myoclonus.

## Materials and Methods

Approval for the study was granted by the Local Ethics Committee (2014/06) and it was recorded in the Clinical Trials (https://clinicaltrials.gov/; NCT02186990). The study comprised a total of 90 patients, aged 18-65 years, who were American Society of Anesthesiologist (ASA) I-II risk group who were to undergo elective surgery with endotracheal intubation under general anaesthesia in Ordu University Research and Training Hospital between in May-August 2014. A written informed consent was obtained from all patients. Patients were excluded if they had any allergy to the medications to be used in the study, chronic use of analgesia or sedatives, body mass index (BMI) > 25 kg/m^2^, anticipated difficult intubation (Mallampati 3 and 4), hypertension or cardiovascular disease.

Patients were not administered with any premedication drugs and on admission to the operating room, electrocardiography (ECG), non-invasive blood pressure, peripheral O_2_ saturation (SpO_2_) and end-tidal CO2 monitoring (Mindray, BeneView T8, Shenzhen, P.R. China) were applied. Neuromuscular monitoring was applied with a TOF Watch SX (Organon Ltd, Dublin, Ireland) device with electrodes placed on the ulnar nerve line and evaluation was made from contractions of the adductor pollicis muscle.

Lactated Ringer’s solution infusion was started at 10 mL kg^-1^ via a 20 G venous cannula in the back of the non-dominant hand. Using a computer generated sequence of numbers and a sealed envelope technique, patients were randomly divided into 3 groups: Group P (n = 30) was administered propofol, group E (n = 30) was administered etomidate and group PE (n = 30) was administered etomidate-propofol (etofol) combination for anaesthesia induction.

After administration of 1 mcg kg^-1^ fentanyl to all patients in anaesthesia induction, patients in group P were administered 2.5 mg kg^-1^ propofol (propofol-lipuro 1%, 10 mg mL^-1^, B Braun, Melsungen, Germany), group E, 0.3 mg kg^-1^ etomidate (Hypnomidate 2 mg mL^-1^, Janssen Pharmaceutica NV, Belgium), and group PE, 1.25 mg kg^-1^ propofol and 0.15 mg kg^-1^ etomidate in separate injectors. The medications were given at the rate of 1 mL s^-1^ and in group PE, the venous cannula was washed out with saline for 5 seconds between medications. When loss of consciousness was obtained (eyelash reflex loss), 0.6 mg kg^-1^ rocuronium was administered and when no response was obtained to the train-of-four (TOF) stimulus with the TOF-guard device, the patient was intubated by the orotracheal route. Following the intubation, the patients were ventilated to preserve end-tidal CO2 pressure between 35-40 mm Hg and anaesthesia was maintained with 2% sevoflurane in a 50% O_2_/air mixture.

The measurement times for the parameters of heart rate (HR) and mean arterial pressure (MAP) were T1, basal values; T2, after induction; T3, before intubation; T4, immediately after intubation; and T5, T6, T7, T8, T9 and T10 at 1, 2, 3, 4, 5, and 10 minutes after intubation. All the measurement intervals, ‘rate pressure product’ (RPP) values were calculated. RRP is defined as the product of systolic blood pressure and HR values.^6^ Values of more than 15 000 mm Hg min^-1^ are accepted as an increased cardiac risk.^7^

In all patients, injection pain was evaluated by the same researcher (NT) by measurement on a 4-point scale as described in previous studies: 0 = no pain, 1 = verbal complaint of pain, 2 = withdrawal of the arm, 3 = both verbal complaint and withdrawal of the arm.^8^ Myoclonus was evaluated in all patients by the same researcher (NT) using the presence of muscular activity: 0 = no myoclonus, 1 = myoclonus present.

### Power Analysis

According to the evaluation made on the basis of a previous study,^9^ when MAP at the first minute of intubation is taken as the main result, in the event of at least 30 patients in each group, it was calculated that in respect of haemodynamic parameters, a 10% difference could be determined between the groups at 80% power and 5% significance (α = 0.05, β = 0.80) (Minitab 13.1 Inc. State College PA, USA).

### Statistical Analysis

Data obtained in the study were analysed with SPSS 16.0 (IBM SPSS Statistics, Chicago, IL, USA). Descriptive statistics were stated as mean ± standard deviation for continuous variables and as number and percentage for nominal variables. Distribution analysis was made with the Kolmogorov-Smirnov test. Age, body mass index (BMI), HR, MAP and RPP were evaluated with one-way analysis of variance (ANOVA) and post-hoc tests with Bonferroni correction. The chi-square test was used for categorical data such as gender, ASA physical status, injection pain and myoclonus. A value of *P  *≤  0.05 was accepted as statistically significant.

## Results

**Figure 1:**
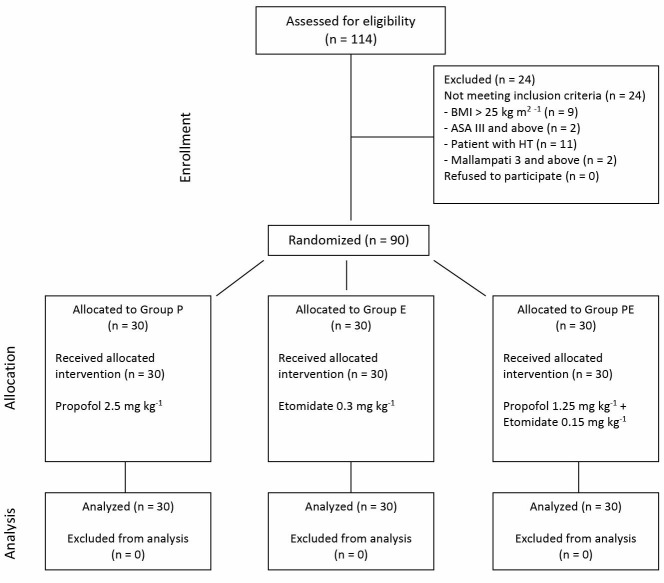
CONSORT Flow Diagram of This Randomized Trial.^10^

The data of 90 patients were evaluated in the study (Figure 1). No statistically significant difference was determined between the groups in respect of patient characteristics and ASA scores (*P *> 0.05; Table 1).

**Table 1:** Patients Characteristics

	**Group P** **(Propofol)** **n = 30**	**Group E** **(Etomidate)** **n = 30**	**Group PE** **(Etofol)** **n = 30**	*** P ***
Age (y)	39.4 ± 14.8	41.4 ± 14.4	38.5 ± 13.5	0.698
BMI (kg/m^ 2 ^)	22.6 ± 2.3	21.8 ± 2.3	23.1 ± 2.0	0.094
Gender (F/M)	18/12	16/14	19/11	0.725
ASA I/II	24/6	21/9	22/8	0.664

Abbreviations: BMI, body mass index; F/M, Female/Male; ASA, American Society of Anesthesiologist. Data are presented as mean ± SD or frequencies.

**Figure 2:**
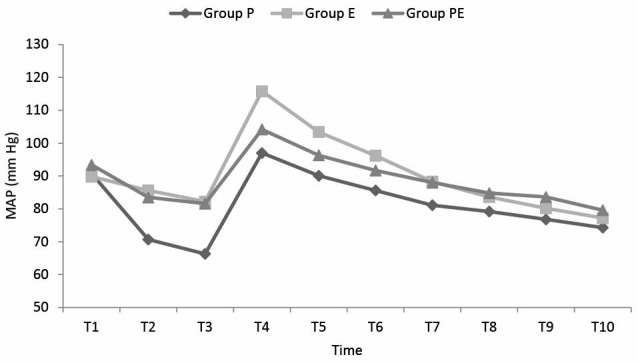
Comparison of mean arterial blood pressure (MAP, mm Hg) between groups. T1: baseline, T2: after the induction, T3: before the intubation, T4: immediately after the intubation and then T5, T6, T7, T8, T9 and T10 for 1, 2, 3, 4, 5 and 10 minutes after the intubation.

The changes in the MAP values according to the times are shown in Figure 2. The MAP values in all 3 groups at T2 and T3 after induction were statistically significantly lower compared to the basal values. In comparison between the MAP values of the groups at T2 and T3, in group P were determined to be statistically significantly lower compared to group E, (*P *< 0.001) and group PE (*P *< 0.01).

At the T4, MAP values of all groups were significantly increased compared to the basal values. In the comparison between the groups at T4, the MAP values of the group E were statistically significantly higher than those of group P (*P *< 0.001) and group PE, (*P *< 0.01).

In group P, MAP values were found to be significantly lower than those of group E at T5, T6 and T7 (*P *< 0.01, 0.01, 0.05 respectively) and of group PE at T7 and T9 (*P *< 0.05 and *P *< 0.05).

**Figure 3:**
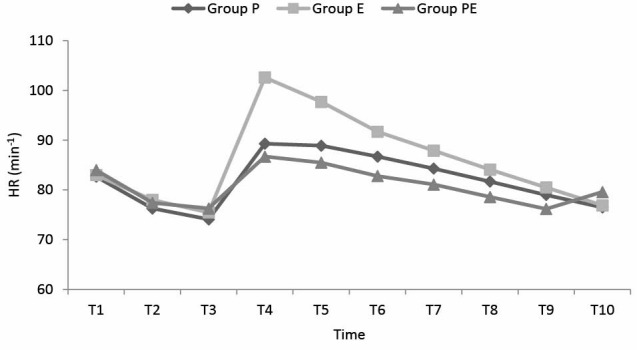
Comparison of heart rate (HR, min^-1^) between groups. T1: baseline, T2: after the induction, T3: before the intubation, T4: immediately after the intubation and then T5, T6, T7, T8, T9 and T10 for 1, 2, 3, 4, 5 and 10 minutes after the intubation.

The changes in the HR values according to the measurement times are shown in Figure 3. In the comparison between the groups, the HR values of the group E at T4 and T5 were determined to be statistically significantly higher than those of Group P and PE (for both groups *P *< 0.01 at T4, and *P *< 0.05 at T5).

**Figure 4:**
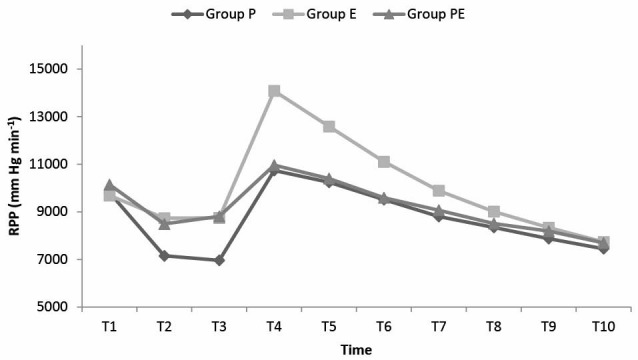
Comparison of rate pressure product (RPP, mm Hg min^-1^) between groups. T1: baseline, T2: after the induction, T3: before the intubation, T4: immediately after the intubation and then T5, T6, T7, T8, T9 and T10 for 1, 2, 3, 4, 5 and 10 minutes after the intubation.

The RPP values according to the measurement times are shown in Figure 4. In the comparison between the groups, the RPP values of group P at T2 and T3 were found to be statistically significantly lower compared to group E and PE (at T2 *P *< 0.01 for group E, *P *< 0.05 for group PE; at T3, *P *< 0.01 for group E and PE). At T4, T5 and T6, the RPP values of the group E were found to be significantly higher compared to those of group P and PE (*P *< 0.01). The number of patients with an RPP value over 15000 mm Hg min^-1^ was 1 (3%) in the group P, 7 (23%) in group E and 2 (7%) in group PE. The difference between the propofol and etomidate groups was statistically significant (*P *< 0.05).

No statistically significant difference was determined between the groups in respect of injection pain. A significant difference was determined between group P and group E in terms of myoclonus incidence (*P *< 0.05; Table 2).

**Table 2:** Incidence of Injection Pain and Myoclonus in Groups

	**Group P** **(propofol)** **n = 30**	**Group E** **(etomidate)** **n = 30**	**Group PE** **(etofol)** **n = 30**
Injection pain			
0	22 (73%)	26 (87%)	27 (90%)
1	2 (7% )	0 (0%)	2 (7%)
2	6 (20%)	4 (13%)	1 (3%)
3	0 (0%)	0 (0%)	0 (0%)
Myoclonus			
0	30 (100%)	24 (80%)	28 (91%)
1	0 (0%)	6 (20%)*	2 (9%)

Data are presented as frequencies (%).

## Discussion

The results of this study in which haemodynamic response to anaesthesia induction and tracheal intubation was evaluated with the use of propofol, etomidate and a combination of these two drugs, demonstrated that a more stable haemodynamic condition was obtained with the drug combination.

In recent years, combinations of various anaesthetic medications, which have created separate beneficial sedative, amnestic and hypnotic effects, have been used in anaesthesia induction. With this method there has been an evident reduction in anaesthetic medication and associated with that, significant reductions in side-effects and costs.^11,12^

Etomidate is one of the iv anaesthetics used in anaesthesia induction, either alone or in combination with other anaesthetic drugs.^13^ In a study by Hosseinzadeh et al,^14^ comparing haemodynamic changes during placement of the laryngeal mask airway (LMA) using propofol, etomidate and a etomidate-propofol combination, after the administration of 2 mcg kg^-1^ iv fentanyl, one group was given 2.5 mg kg^-1^ propofol, one group, 0.3 mg kg^-1^ etomidate, and one group, 1 mg kg^-1^ propofol + 0.1 mg kg^-1^ etomidate. LMA placement was made after the loss of eyelash reflex and no response to verbal commands. The main finding of the study was that more stable haemodynamics were provided by etofol compared to propofol and etomidate. Although the doses of both drugs are reduced in the etomidate-propofol combination, it was reported that a more stable haemodynamic state and better conditions for LMA placement were provided.^14^

In a study by Saricaoglu et al,^15^ haemodynamics, myoclonus and injection pain were evaluated with propofol and etomidate at doses of the same bispectral index value as etomidate-propofol combination in the same injector in anaesthesia induction. The etomidate-propofol combination in that study was provided at a 1:1 ratio of 1% propofol (20 mg mL^-1^) and etomidate (2 mg mL^-1^). In the anesthesia induction, the medications were applied by titration to provide at a target BIS value of 40. From the results of the study, it was reported that anaesthesia induction with a combination of etomidate and propofol provided a pain-free injection, lower rate of myoclonus, and in comparison with propofol and etomidate used alone, achieved a quicker induction and better haemodynamic stability.

In cardiac and non-cardiac surgery, there is evidence related to undesired results during general anaesthesia independent of hypo or hypertension in the patient.^16,17^ In a study of 4096 patients in which hypotension following general anaesthesia induction was evaluated, risk factors for hypotension were defined as ASA III-IV risk group, age of ≥50 years, baseline MAP of <70 mm Hg, the use of propofol for anaesthesia induction and the use of a high dose of fentanyl in induction. To avoid severe hypotension in patients aged over 50 years with ASA III risk scores or more, an alternative to propofol should be considered in anaesthesia induction and it was particularly recommended that propofol is avoided in cases with baseline MAP of <70 mm Hg.^18^

In a study comparing propofol+ketamine and propofol+etomidate combinations in elderly patients, it was found that as the application of propofol prevented haemodynamic changes, both ketamine and etomidate were equally effective.^19^

Besides providing good cardiovascular stability, etomidate may also cause nausea and vomiting, injection pain, myoclonus and side-effects on the endocrine system.^3^ Etomidate reversibly inhibits 11-β-hydroxylase and prevents the return of 11-deoxycortisol to cortisol. Single-dose etomidate inhibits 11-β-hydroxylase in 5-8 hours postoperatively.^20^ Another significant disadvantage of etomidate is that it has no analgesic effect, it cannot effectively reduce the temporary sympathetic response to endotracheal intubation.^21,22^ This situation may cause a short but uncomfortable attack of hypertension and tachycardia. Even though this hyperdynamic response is short, it causes an increase in intracranial pressure and increased myocardial workload.^23^ In addition to these known side-effects of etomidate, it has been reported that occasionally ventricular tachycardia and fibrillation may be seen.^24^

In a study by Möller et al^25^ which used propofol and etomidate in general anaesthesia induction accompanied by BIS monitoring, the MAP, cardiac index (CI) and systemic vascular resistance index (SVRI) values of 48 patients were compared. The haemodynamic data were found to be higher in the etomidate group up to 7 minutes after intubation. A significantly high level of hypotension incidence was found in the propofol group and a significantly high level of hypertension incidence in the etomidate group. Compared with etomidate, the use of propofol was determined to have caused less hypertension and tachycardia after intubation. In the current study, the MAP values after induction in the propofol group were significantly lower than those of the other two groups. Following intubation, the MAP and HR values of the etomidate group were statistically significantly higher than those of the other two groups. These results confirm with those in literature.

In another study comparing the use of propofol and etomidate in anaesthesia induction, it was reported that when opioid and/or benzodiazepine support is not suitable because of a negative cardiovascular profile, myoclonus and poor mask ventilation, etomidate may be suitable in induction.^26^

Another study reported that after anaesthesia induction with etomidate (0.3 mg kg^-1^), the ideal fentanyl dose was 5-10 mcg kg^-1^ to prevent a haemodynamic response to laryngoscopy and intubation.^27^ However, it can be predicted that the use of such a high dose of fentanyl may cause increased hypotension and nausea and vomiting.

In a study by Muriel et al,^28^ a comparison was made of propofol (2 mg kg^-1^), thiopental (5 mg kg^-1^) and etomidate (0.3 mg kg^-1^) in anaesthesia induction. A statistically significant increase was determined in systolic and diastolic arterial pressure and HR in the etomidate and thiopental groups after intubation and the highest rates of complications were reported in the etomidate group.

In two studies which compared propofol, thiopental and etomidate induction in intubation without muscle relaxant, as appropriate conditions could not be provided in the etomidate group, the study was prematurely terminated.^29,30^

In another study, the haemodynamic response to orotracheal intubation was evaluated following anaesthesia induction with midazolam and etomidate. Although the systolic and diastolic pressures and RPP values were found to be lower in the midazolam group, it was reported that neither of the induction agents could prevent the haemodynamic response to intubation.^31^

It is known that RPP is an indicator of myocardial stress and maximal oxygen consumption and is the best indirect measurement method of myocardial oxygen consumption.^6^ In the current study, the RPP values of the etomidate group after intubation were statistically significantly higher than those of the other two groups. This was evaluated as an indicator that the sympathetic response formed after laryngoscopy and intubation could not be adequately prevented with etomidate.

In patients not receiving premedication in anaesthesia induction with etomidate, myoclonus incidence has been reported at 50%-80%.^32^ In the current study, myoclonus was determined at 20% in group E and 9% in group PE. These low rates of myoclonus are thought to be due to premedication with fentanyl. Previous studies have reported the incidence of myoclonus with fentanyl use at 8% to 40%.^21,33^

Injection pain is a significant clinical problem in both propofol and etomidate use. In the current study, the incidence of injection pain was 27% in group P, 13% in group E and 10% in group PE, with no statistically significant difference between these rates. In literature, propofol injection pain has been reported at rates of 40%-86%.^34^ With fentanyl premedication, rates such as 40%, 19% and 8% have been reported.^35-37^ Reported rates of 50%-60% of etomidate injection pain are also reduced with fentanyl premedication.^21^

In a study by Saricaoglu et al^15^ comparing propofol, etomidate and etofol in anaesthesia induction, injection pain in the etofol group was found to be lower than in the other two groups and myoclonus incidence was lower than in the etomidate group. The incidence of injection pain was reported as 83.8% in the propofol group and as 63.2% in the etomidate group. Myoclonus incidence was determined as 93.4% in the etomidate group. The reason for the high rates of these results compared to the results of the current study is thought to be that no premedication was administered in the Saricaoglu study.

In literature, propofol and etomidate mixed in the same injector have been used.^15^ Due to the risk of propofol contamination in particular, it has been reported that it is necessary to apply strict aseptic techniques during preparation and application.^38^ Severe infection tables have been reported because of propofol contamination.^38,39^ In the current study, separate injectors were used because of the increased risk of contamination while preparing the combination.

Limitations of the current study were primarily that BIS measurement was not applied to evaluate loss of consciousness and to determine the depth of anaesthesia. It has been reported that for etomidate, a BIS value of 50 is sufficient for conscious movement and absence of memory in tracheal intubation.^40^ In a study by Saricaoglu et al^15^ propofol, etomidate and etofol were administered in a manner that the BIS value would be 40. The doses used in the current study were similar to those used in the Saricaoglu study.

Another limitation of the current study is that plasma cortisol and adrenocorticotropic hormone level were not measured. However, as the use of single-dose etomidate causes adrenocortical suppression, it has been reported to be temporary and not of clinical importance.^20^

In the literature there are 3 studies on the etomidate-propofol combination. The first of these is the study by Sarıcaoglu et al. comparing propofol, etomidate and etofol in terms of effects on hemodynamic changes only during anesthesia induction. Another is a study comparing effects of etomidate and ketamine added to propofol by Hosseinzadeh et al. The final study is again by Hosseinzadeh et al and compares propofol, etomidate and etofol in terms of hemodynamic changes during LMA insertion. Our study compared etofol with etomidate and propofol, not only during anesthesia induction but after tracheal intubation. In conclusion, with the etomidate-propofol combination, a milder haemodynamic response was determined compared to propofol and etomidate used alone, both at anaesthesia induction and after the intubation. Etofol can be a valuable alternative in patients where cardiovascular fluctuations are not wanted.

## Ethical issues

Ethics committee approval was received for this study from Ethics Committee of Harran University (2014/06). Also, written informed consent was obtained from patients who participated in this study.

## Competing interests

Authors declare no conflict of interest in this study.
